# Associated lifestyle factors of elevated plasma aldosterone concentration in community population, gender-stratified analysis of a cross-sectional survey

**DOI:** 10.1186/s12889-024-18796-0

**Published:** 2024-05-22

**Authors:** Adalaiti Maitituersun, Mulalibieke Heizhati, Nanfang Li, Lin Gan, Mei Li, Ling Yao, Wenbo Yang, Shasha Liu, Xiayire Aierken, Hui Wang, Miaomiao Liu, Jing Hong, Ting Wu, Delian Zhang, Qing Zhu

**Affiliations:** Hypertension Center of People’s Hospital of Xinjiang Uygur Autonomous Region; Xinjiang Hypertension Institute; NHC Key Laboratory of Hypertension Clinical Research; Key Laboratory of Xinjiang Uygur Autonomous Region “Hypertension Research Laboratory”; Xinjiang Clinical Medical Research Center for Hypertension (Cardio-Cerebrovascular) Diseases, No. 91 Tianchi Road, Urumqi, 830001 Xinjiang China

**Keywords:** Plasma aldosterone, Physical activity, Sleep quality and sleep disordered breathing, Depression and anxiety, Blood pressure

## Abstract

**Background:**

Aldosterone plays important parts in development of cardio-metabolic diseases as end product of renin-angiotensin-aldosterone system. However, factors elevating circulating aldosterone are not clear, and lifestyle-related factors are suggested to be involved, whereas less studied. Therefore, we aimed to explore the association of lifestyle factors with plasma aldosterone concentration (PAC) in community population.

**Methods:**

In this cross-sectional study, we recruited participants using multistage random sampling from Emin China in 2019, and collected data and fasting blood samples. The considered lifestyle factors included obesity parameters (neck circumference, abdominal circumference), alcohol consumption, blood pressure (BP), physical activity, sleep duration, sleep quality, mental state (depression and anxiety), fasting blood glucose (FBG), and lipid profiles (total cholesterol and triglyceride). PAC was measured using radioimmunoassay. We performed sex-stratified linear and logistic regressions to explore associated factors of PAC. Component analysis was further performed to identify the main factors affecting PAC.

**Results:**

Twenty-seven thousand four hundred thirty-six participants with 47.1% men were included. Obesity parameters (neck circumference, abdominal circumference), glucose metabolism (FBG), psychological status (anxiety status in men and women, depression status in men), BP, liver function (in men), lipid metabolism (TC and TG in men), sleep parameters (sleep quality in women), and renal function (in women) are the main factors associated with elevated PAC.

**Conclusion:**

lower physical activity, alcohol consumption, higher BP, fat accumulation, dyslipidemia, higher fasting blood glucose, and presence of depression and anxiety were the main factors associated with eleveated PAC.

**Supplementary Information:**

The online version contains supplementary material available at 10.1186/s12889-024-18796-0.

## Introduction

Aldosterone, a member of renin-angiotensin-aldosterone system (RAAS), is the major mineralocorticoid involved in the regulation of water, electrolyte and blood pressure (BP) homeostasis [1]. Aldosterone is essential for life but damaging to the vascular endothelium when dysregulated [2].

In recent years, aldosterone has gained significant attention as one of the contributors to cardio-metabolic risk factors such as diabetes, hypertension, and obesity [3], chronic kidney disease (CKD) [4] and cardiovascular morbidity and mortality in various population [5–8]. The background pathogenesis may include direct and indirect aldosterone-mediated adverse effects on vascular system, such as oxidative stress, inflammation, hypertrophic remodeling, fibrosis, and endothelial dysfunction [5].

Regulation of aldosterone synthesis and secretion is mainly influenced by stimulants such as adrenocorticotropic hormone, circulating potassium concentration and angiotensin II [9, 10]. However, recent studies have shown life style related factors may also influence circulating aldosterone levels. For instance, a population-based study reported that, circulating glucose, BP, body mass index (BMI), smoking status, and total cholesterol are associated with elevation in circulating aldosterone concentration [11]. Evidence also suggests that presence of depression and or anxiety disorders are relevant to elevated aldosterone in the absence of changes in renin or cortisol concentrations [12, 13]. In addition, whether some other lifestyle factors are associated with aldosterone is unclear or controversial. For example, physical activity in the form of aerobic exercise is associated with lower aldosterone in a study [14], whereas not in others [15, 16]. Moreover, some other factors or confounders that may influence aldosterone, such as sleep quality, sleep disordered breathing (SDB), and or medications, may not have been adequately considered in previous studies.

Based on the existing evidence, it might be reasonable to speculate that circulating aldosterone is influenced by life-style associated factors. Also, understanding the reasons or factors associated with elevation in circulating aldosterone might be of important significance and may provide us with clues to lower its circulating concentration, when considering the adverse effects of elevated aldosteron in circulation on health, and when considering the cardio-renal protective effects of mineralocorticoid receptor antagonists (MRAs) [17, 18] and of emerging aldosterone inhibitors [19, 20].

Therefore, the aim of this study was to analyze the factors associated with elevated plasma aldosterone concentration (PAC) in community based population [21, 22] by considering sex, since several studies have observed gender differences in levels and or regulators of aldosterone between men and women [5].

### Study Population

As described previously [21, 22], we used a multi-stage stratified sampling method to enroll study participants aged ≥ 18 years. At the first stage, Emin county was divided into three settings as urban, agricultural and stock-raising areas [21]. At the second stage, two corresponding streets or villages were randomly selected using sample random sampling (SRS) method. At the third stage, subjects aged ≥ 18 years were selected using SRS method.

**Fig. 1:**
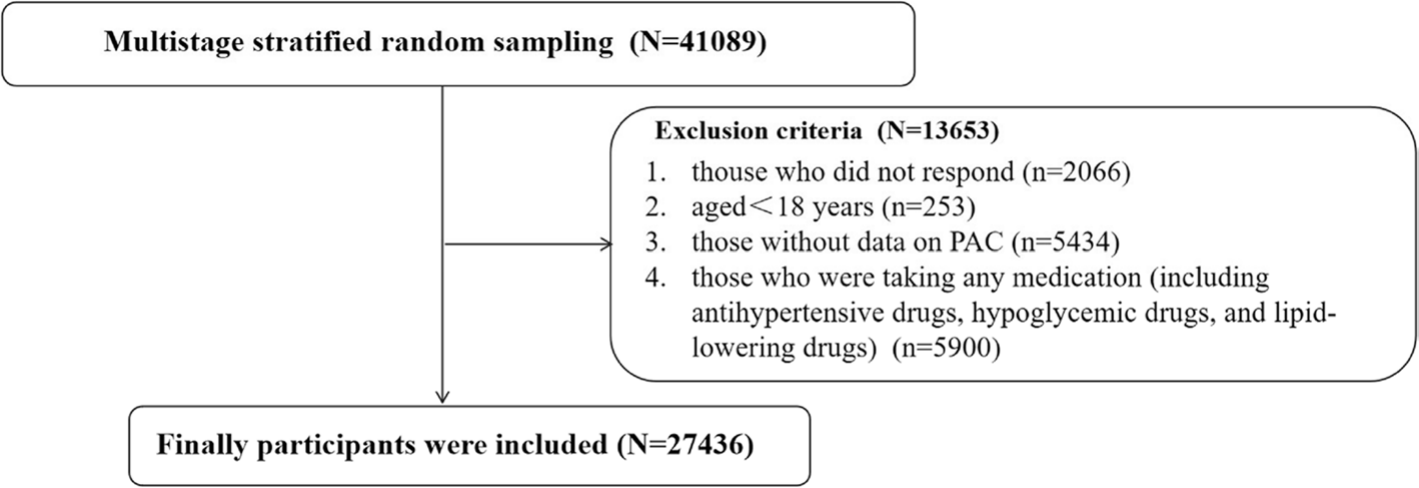
Flowchart for study population

Inclusion criteria: 1) those who had lived at their current address for ≥ 6 months, and 2) those who agreed to participate in the survey and signed an informed consent form. Exclusion criteria: 1) those who were unable to cooperate with the survey due to mental, hearing, mental and or other problems, 2) those without data on PAC and 3) those who were taking any medication (including antihypertensive, hypoglycemic, and or lipid-lowering medications) for the current analysis as given in Fig. 1.

Ethics’ Committee of People’s Hospital of Xinjiang Uygur Autonomous Region approved the study protocol, and all the participants signed informed consent.

### Data collection

Questionnaires were completed for each participant by face-to-face interview with trained investigators including sociodemographic characteristics (age, gender, occupation, and education), cigarette and alcohol consumption, global physical activity questionnaire (GPAQ), Pittsburgh sleep quality index (PSQI), No-SAS scale, Zung’s self rating anxiety and depression scales (SAS, SDS), and medical histories (hypertension, dyslipidemia, diabetes, coronary heart disease and stroke including medicine intake) [21].

### Measurements

Height, weight, neck and abdominal circumference, and BP were measured. BP was measured three times with a Professional Portable Blood Pressure Monitor (OMRON HBP-1300, Kyoto, Japan) on the right arm positioned at heart level after the participant was sitting at rest for 5 min, with 30 s between each measurement. Average of three values was used. BMI was calculated as weight (kg) /height (m^2^).

Fasting venous blood samples were collected, and centrifugated onsite. Some blood samples were tested for concentrations of serum creatinine, fasting blood glucose (FBG), triglycerides (TG), total cholesterol (TC) and alanine aminotransferase (ALT) and aspartate aminotransferase (AST) [21, 22] at local hospitals. Some part of the sample was transported to Xinjiang Hypertension Institute (located in Urumqi, 500–600 km in distance) in portable refrigerators and were stored at−70 °C until measurement in 2021. PAC was measured using radioimmunoassay (DSL−8600 ACTIVE Aldosterone Coated Tube Radioimmunoassay Kit; Diagnostic Systems Laboratories, Webster, TX, USA) with the intra- and inter-assay coefficients of variation of 5.6% and 8.5% in both data. The details of the measurements are in accordance with our previous studies [22].

### Definitions

Age is categorized into young (< 45 years), middle-aged (45–60 years), and elderly (≥ 60 years). Education attainment status was categorized into primary and lower, junior high, senior high and higher [21]. Occupation was categorized as manual and mental work [21]. Current cigarette consumption is defined as smoking at the time of survey, exposure to secondhand smoking and the year quitting smoking ≤ 5 years, non-smoking is defined as never smoking and the year of quitting smoking ≥ 5 years. Alcohol consumption is defined as consuming an alcoholic beverage at least once per week in the past month [21]. Physical activity was assessed using Chinese version of GPAQ [23], and classified into high (≥ 3000 MET-minutes per week from any combination of walking, moderate or vigorous activity, or ≥ 1500 MET-minutes per week from high intensity activity), medium (not meeting the criteria for the high category but achieving ≥ 600 MET-minutes per week), and low (not meeting any of above criteria) levels [24]. Sleep quality was assessed with PSQI [25], in which global score ranges from 0 to 21 and score ≥ 6 indicates poor sleep quality [26]. Sleepduration was divided into three criteria as normal sleep (6–8 h), shorter sleep (< 6 h) and longer sleep (> 8 h). No-SAS score, including neck circumference, BMI, snoring, age and gender and ranging from 0 to 17, was used to screen SDB and SDB was defined if the No-SAS score was ≥ 8 points [22]. Anxiety and depression status are defined as standardized SAS score ≥ 45 and SDS score ≥ 50. Hypertension is defined as systolic BP ≥ 140 mmHg, and/or diastolic BP ≥ 90 mmHg, and/or use of anti-hypertensive medicine within 2 weeks. Diabetes is defined as FBG ≥ 7.0 mmol/L, and/or self-reported previous diagnosis by physicians and/or intake of hypoglycemic agents within past 2 weeks. Cardiovascular disease is defined as self-reported medical history of coronary heart disease and stroke. CKD is defined as glomerular filtration rate < 60 mL/min/1.73m2. Neck circumference was divided into larger and normal ones by 40cm and 35cm as the cutoff for men and women respectively [27]. Abdominal obesity is defined as abdominal circumference ≥ 90 cm for men and ≥ 85 cm for women.

### Statistical analysis

Characteristics of total, men and women participants were described using descriptive statistics. Data were expressed as mean ± standard deviation or median (quartiles), and counts were expressed as percentages (%). All data were tested for normality and chi-square before analysis.

Linear and logistic regression analyses were used to explore the associated factors of PAC. In linear regression, parameters including PAC with skewed distribution were log transformed. In logistic regression analysis, PAC was categorized into higher and lower median group by the median, (13.06 ng/dl, 15.38 ng/dl for men and women respectively), of PAC and lower median group was set as reference. Results were expressed as regression coefficients (B value) or odds ratios (OR) and 95% confidence intervals (95% CI). Univariate linear or logistic regression models were performed for variable selection to be adjusted. Tolerance and variance inflation factor (VIF) were examined to identify multicollinearity, and multicollinearity is a concern if VIF is > 10 and tolerance is < 0.10.

Principal component analysis was performed to screen out the main associated factors of PAC, in which statistically significant variables (*P* < 0.05) in the results of the linear and logistic regression analysis were included in the principal component analysis.

Stratified analysis was performed for regression analysis in population with hypertension and or diabetes, populations without hypertension and diabetes, and populations without hypertension and diabetes and SDB.

SPSS 25.0 software was applied for statistical analysis of the data. The test level was set at ɑ = 0.05 and a two-sided *P* value < 0.05 was considered statistically different.

## Results

### Baseline population characteristics

In total 27,436 participants with PAC data were included, with 12,933 (47.1%) men as in Table 1.

**Table 1:** General characteristics of total and sex-specific study population

Characteristics	Men (*n* = 12,933)	Women (*n* = 14,503)	Total (*n* = 27,436)
Age (years)	45 (35, 54)	45 (34, 53)	45 (35, 54)
Body mass index (kg/m^2^)	25.28 (22.72, 27.98)	24.69 (22.07, 27.68)	24.98 (22.36, 27.85)
Body mass index ≥ 28 kg/m^2^ (n,%)	3213 (24.9)	3321 (22.9)	6534 (23.81)
Neck circumference, cm	37.0 (36.0, 40,0)	33.1 (31.6, 35.0)	35.2 (32.9, 38.0)
Abdominal circumference, cm	90.4 (82.7, 97.5)	84.1 (77.0, 92.4)	87.2 (79.3, 95.2)
Abdominal obesity (n,%)	5898 (46.7)	5507 (39.0)	11,405 (41.57)
Education, primary and lower (n,%)	3640 (29.0)	4221 (30.1)	7861 (28.65)
junior high	5315 (42.4)	5007 (35.7)	10,322 (37.62)
senior high and higher	3578 (28.5)	4794 (34.2)	8372 (30.51)
Occupation, manual (n,%)	7419 (60.2)	8406 (61.0)	15,825 (57.68)
Cigarette consumption (n,%)	7446 (60.4)	2122 (14.6)	9568 (34.87)
Alcohol intake (n,%)	7122 (58.0)	1224 (8.4)	8436 (30.75)
Systolic blood pressure (mmHg)	125 (115, 137)	117 (1.08, 130)	120 (110, 133)
Diastolic blood pressure (mmHg)	80 (72, 89)	75 (69, 82)	77 (70, 85)
Total MET-minutes	3360 (840, 10,080)	2610 (554, 7260)	3360 (720, 8400)
Physical activity intensity, high (n,%)	7288 (64.0)	7151 (56.1)	14,439 (52.63)
middle (n,%)	2575 (22.6)	3704 (29.0)	6279 (22.89)
Pittsburgh sleep quality index	3.0 (1.0, 5.0)	4.0 (2.0, 7.0)	3.0 (1.0, 6.0)
Poor sleep quality (n,%)	2982 (23.7)	4601 (32.6)	7583 (27.64)
Sleep duration (h)	7.0 (6.0, 8.0)	7.0 (6.0, 8.0)	7.0 (6.0, 8.0)
<6 h (n,%)	1636 (14.7)	2075 (16.7)	3711 (13.53)
>8 h (n,%)	4264 (38.2)	1365 (10.9)	5629 (20.51)
No SAS score	6.0 (2.0, 9.0)	3.0 (0.0, 5.0)	4.0 (2.0, 7.0)
Sleep disordered breathing (n,%)	4155 (36.7)	936 (7.1)	5091 (18.56)
Depression status (n,%)	225 (2.1)	623 (5.1)	848 (3.09)
Self rating depression score	28.75 (26.25, 32.50)	31.25 (27.50, 37.50)	30.0 (26.25, 35.0)
Anxiety status (n,%)	376 (3.4)	1099 (8.9)	1475 (5.38)
Self rating anxiety score	27.50 (25.0, 31.25)	28.75 (25.0, 35.0)	28.75 (25.0, 33.75)
Hypertension (n,%)	4095 (33.7)	2266 (17.4)	6361 (23.18)
Diabetes (n,%)	948 (8.3)	779 (6.2)	1727 (6.29)
Cardiovascular disease (n,%)	150 (1.3)	203 (1.6)	353 (1.29)
Dyslipidemia (n,%)	3583 (31.4)	2979 (23.7)	6562 (23.92)
Fasting blood glucose (mmol/l)	5.28 (4.79, 5.82)	5.20 (4.73, 5.70)	5.24 (4.76, 5.77)
Alanine aminotransferase (µ/l)	22.0 (17.0, 30.0)	19.0 (14.0, 25.0)	20.0 (15.0, 28.0)
Aspartate aminotransferase (µ/l)	22.0 (18.0, 27.0)	20.0 (17.0, 25.0)	21.0 (18.0, 26.0)
Creatinine (umol /l )	73.2 (63.5, 84.6)	63.3 (53.6, 77.2)	68.6 (57.1, 80.7)
Renal insufficiency (n,%)	182 (1.5)	764 (5.7)	946 (3.45)
Total cholesterol (mg/dl)	4.7 (4.0, 5.5)	4.6 (3.9, 5.4)	4.66 (3.97, 5.42)
Triglyceride (mg/dl)	1.3 (0.9, 1.8)	1.1 (0.8, 1.6)	1.2 (0.8, 1.7)
Plasma aldosterone concentration (ng/dl)	13.06 (9.19, 18.69)	15.38 (10.94, 22.16)	14.24 (10.12, 20.41)

### Linear and logistic regression models

**Fig. 2:**
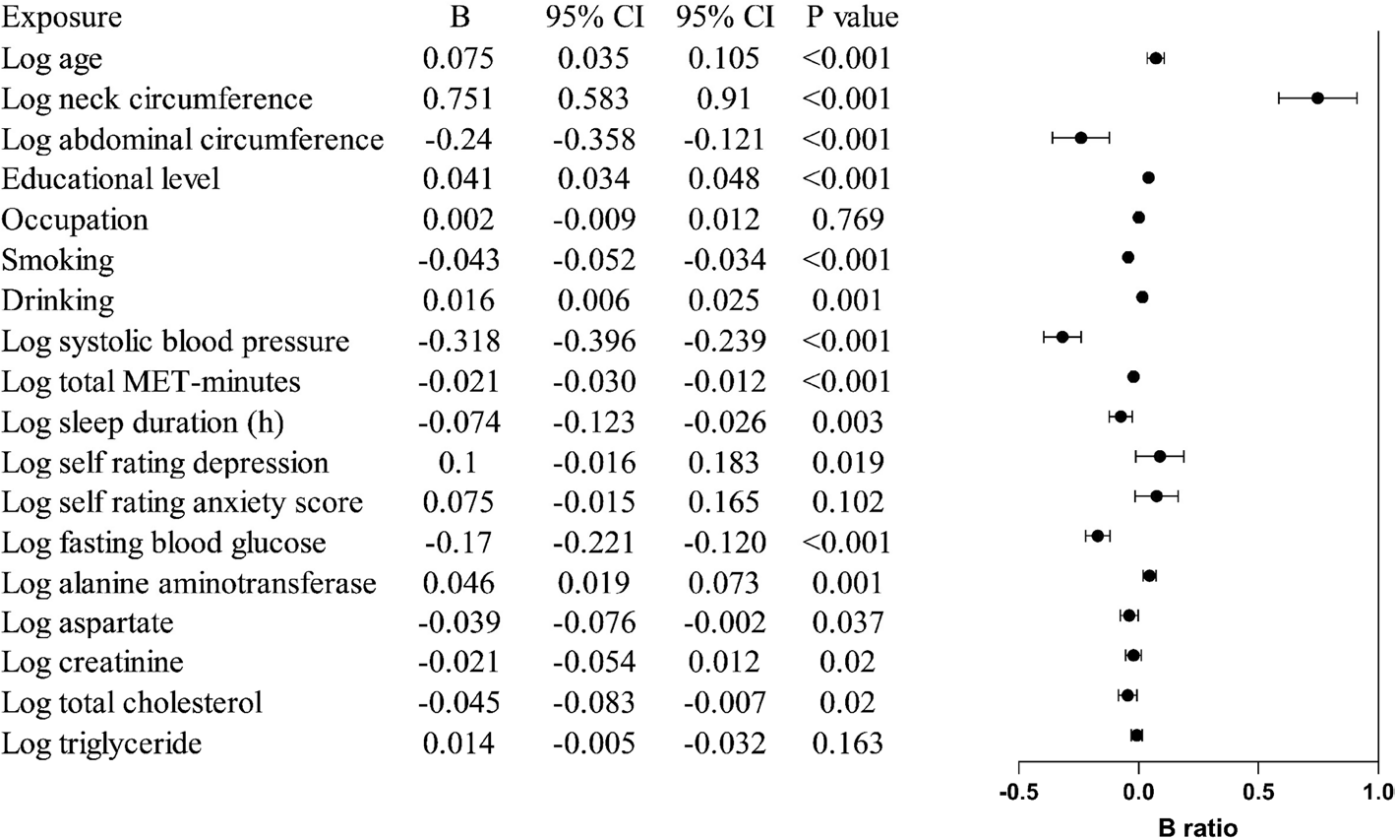
Multi-variable linear regression for PAC in study population in men

**Fig. 3:**
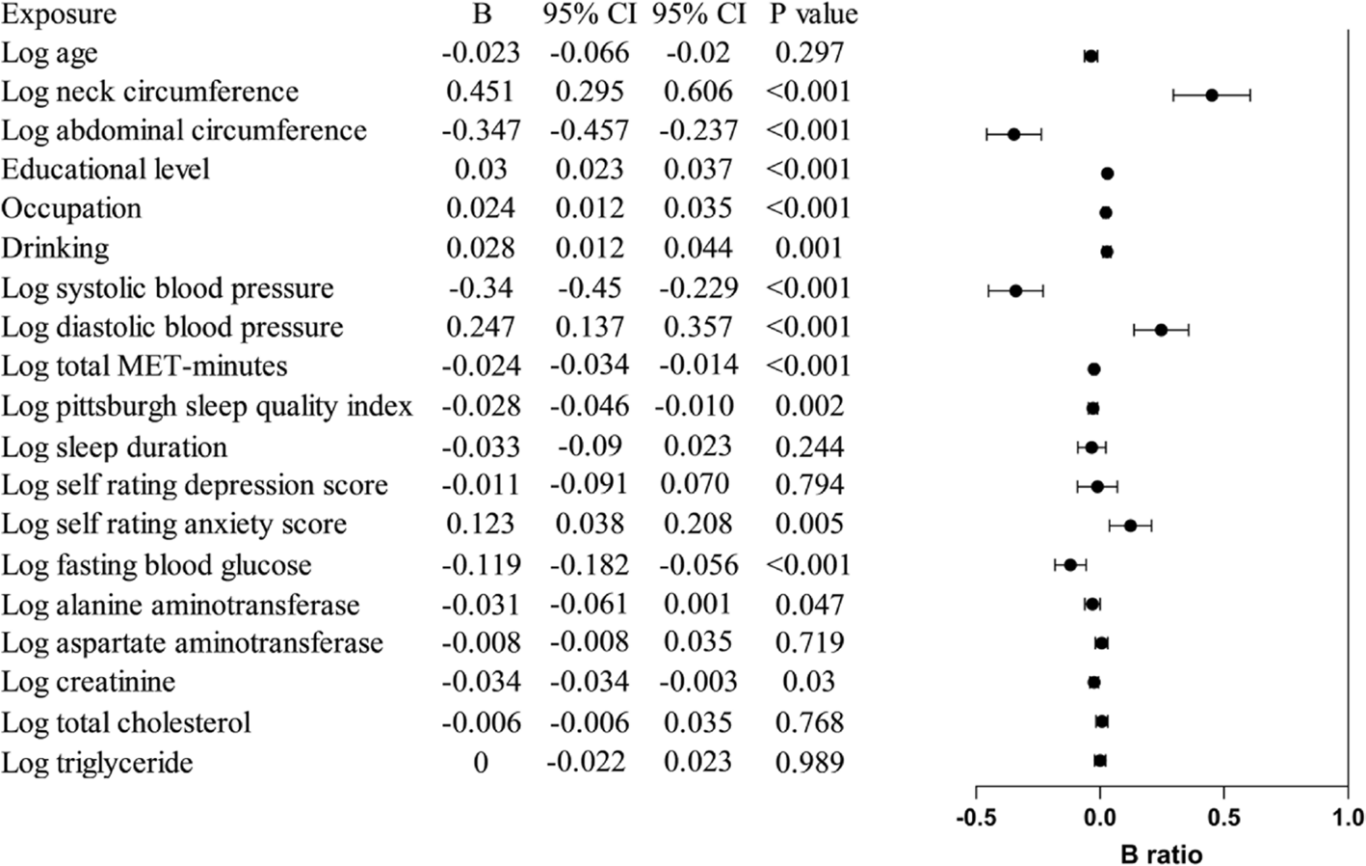
Multi-variable linear regression for PAC in study population in women

Linear regression analysis showed that age, neck circumference, education status, alcohol consumption, SDS score, and ALT were positively associated with log-PAC in men; neck circumference, education status, alcohol consumption, diastolic BP (DBP), and SAS score were positively associated with log-PAC in women. Physical activity was negatively associated with PAC in both gender *(P* for all < 0.05) (Table 2; Figs. 2 and 3).

**Table 2:** Multivariate linear regression analysis between parameters and log plasma aldosterone concentration in sex-specific population (B, 95%CI, P)

	Men	Women
Age	0.075 (0.035, 0.105), < 0.001	−0.023 (−0.066,−0.020), 0.297
Neck circumference	0.751 (0.583, 0.910), < 0.001	0.451 (0.295, 0.606), < 0.001
Abdominal circumference	−0.240 (−0.358,−0.121), < 0.001	−0.347 (−0.457,−0.237), < 0.001
Educational level	0.041 (0.034, 0.048), < 0.001	0.030 (0.023, 0.037), < 0.001
Occupation	0.002 (−0.009, 0.012), 0.769	0.024 (0.012, 0.035), < 0.001
Smoking	−0.043 (−0.052,−0.034), < 0.001	/
Drinking	0.016 (0.006, 0.025), 0.001	0.028 (0.012, 0.044), 0.001
Systolic blood pressure	−0.318 (−0.396,−0.239), < 0.001	−0.340 (−0.450,−0.229), < 0.001
Diastolic blood pressure	/	0.247 (0.137, 0.357), < 0.001
Total MET-minutes	−0.021 (−0.030,−0.012), < 0.001	−0.024 (−0.034,−0.014), < 0.001
Pittsburgh sleep quality index	/	−0.028 (−0.046,−0.010), 0.002
Sleep duration	−0.074 (−0.123,−0.026), 0.003	−0.033 (−0.090, 0.023), 0.244
Self rating depression score	0.100 (−0.016, 0.183), 0.019	−0.011 (−0.091, 0.070), 0.794
Self rating anxiety score	0.075 (−0.015, 0.165), 0.102	0.123 (0.038, 0.208), 0.005
Fasting blood glucose	−0.170 (−0.221,−0.120), < 0.001	−0.119 (−0.182,−0.056), < 0.001
Alanine aminotransferase	0.046 (0.019, 0.073), 0.001	−0.031 (−0.061, 0.001), 0.047
Aspartate aminotransferase	−0.039 (−0.076,−0.002), 0.037	−0.008 (−0.051, 0.035), 0.719
Creatinine	−0.021 (−0.054, 0.012), 0.020	−0.034 (−0.064,−0.003), 0.030
Total cholesterol	−0.045 (−0.083,−0.007), 0.020	−0.006 (−0.048, 0.0350, 0.768
Triglyceride	0.014 (−0.005,−0.032), 0.163	0.000 (−0.022, 0.023), 0.989

Logistic regression analysis showed that larger neck circumference (OR = 1.13, 1.03–1.24, *P* < 0.001), abdominal obesity (1.19, 1.09–1.31, *P* < 0.001), higher education attainment (junior high vs. primary and lower: 1.21, 1.10–1.34, *P* < 0.001; senior high and higher vs. primary and lower: 1.87, 1.66–2.09, *P* < 0.001), alcohol consumption (1.12, 1.03–1.22, *P =* 0.009), lower physical activity level (middle vs. high: 1.21, 1.09–1.33, *P* < 0.001; low vs. high: 1.13, 1.00−1.28, *P* = 0.046), and presence of depression (1.68, 1.18–2.38, *P* = 0.004) and anxiety (1.50, 1.15–1.96, *P* = 0.002) were associated with higher PAC in men (Table 3).

**Table 3:** Multivariate logistic regression analysis stratified by sex-specific study population (OR, 95%CI, P)

	Men	Women
< 45 years (vs. > 60 years)	/	1.35 (1.15, 1.57), < 0.001
45–60 years (vs. > 60 years)	/	0.97 (0.97, 1.32), 0.108
Neck circumference (≥ 40 vs. < 40 cm)	1.13 (1.03, 1.24), < 0.001	1.64 (1.18, 2.29), 0.004
Abdominal obesity (yes vs. no)	1.19 (1.09, 1.31), < 0.001	0.88 (0.81, 0.97), 0.007
Junior high (vs. ≤ primary)	1.21 (1.10, 1.34), < 0.001	1.17 (1.05, 1.30), 0.005
≥Senior high (vs. ≤ primary)	1.87 (1.66, 2.09), < 0.001	1.76 (1.46, 1.89), < 0.001
Occupation (intelligent vs. manual)	0.89 (0.81, 0.97), 0.010	1.18 (1.06, 1.30), 0.002
Smoking ( yes vs. no )	0.73 (0.67, 0.79), < 0.001	/
Alcohol intake (yes vs. no)	1.12 (1.03, 1.22), 0.009	1.34 (1.16, 1.56), < 0.001
Physical activity (middle vs. high)	1.21 (1.09, 1.33), < 0.001	1.38 (1.26, 1.53), < 0.001
Physical activity( low vs. high)	1.13 (1.00, 1.28), 0.046	1.19 (1.05, 1.35), 0.008
Sleep quality (poor vs. good)	/	/
Sleep duration (≤ 6 h vs. 6–7 h)	/	/
Sleep duration (> 8 h vs. 6–7 h)	/	/
Depression ( yes vs. no )	1.68 (1.18, 2.38), 0.004	1.40 (1.14, 1.72), 0.001
Anxiety (yes vs. no )	1.50 (1.15, 1.96), 0.002	/
Hypertension ( yes vs. no )	0.80 (0.74, 0.88), < 0.001	/
Diabetes (yes vs. no )	0.83 (0.72, 0.97), 0.017	/
CVD (yes vs. no)	/	/
Renal inadequacy (yes vs. no)	/	/

Younger age (< 45 vs. ≥ 60 years: 1.35, 1.15–1.57, *P* < 0.001, 45–60 vs. ≥ 60 years: 1.13, 0.97–1.32, *P* = 0.108), larger neck circumference (1.64, 1.18–2.29, *P* = 0.004), higher educational attainment (1.17, 1.05–1.30, *P =* 0.005; 1.16, 1.46–1.89, *P* < 0.001), mental work (1.18, 1.06–1.30, *P* = 0.002), alcohol consumption (1.34, 1.16–1.56, *P* < 0.001), lower physical activity (1.38, 1.26–1.53, *P* < 0.001; 1.19, 1.05–1.35,*P* = 0.008), and presence of depression (1.40, 1.14–1.72, *P* < 0.001) were associated with higher PAC in women (Table 3). Selection of variables to be adjusted was given Tables 4 and 5.

**Table 4:** Univariate linear regression analysis between parameters and log plasma aldosterone concentration in sex-specific population (B, 95%CI, P)

	Men	Women
Age	−0.073(−0.091,−0.052), < 0.001	−0.281 (−0.303,−0.256), < 0.001
Neck circumference	0.781 (0.679, 0.889), < 0.001	0.182 (0.071, 0.295), 0.002
Abdominal circumference	0.144 (0.071, 0.214), < 0.001	−0.404 (−0.466,−0.342), < 0.001
Educational level	0.049 (0.044, 0.053), < 0.001	0.052 (0.048, 0.057), < 0.001
Occupation	0.035 (0.028, 0.042), < 0.001	0.061 (0.053, 0.069), < 0.001
Smoking	−0.029 (−0.036,−0.022), < 0.001	0.003 (−0.007, 0.013), 0.537
Drinking	0.016 (0.009, 0.023), < 0.001	0.049 (0.036, 0.062), < 0.001
Systolic blood pressure	−0.26 (−0.32,−0.20), < 0.001	−0.446 (−0.501,−0.390), < 0.001
Diastolic blood pressure	−0.002 (−0.059, 0.054), 0.940	−0.194 (−0.255,−0.134), < 0.001
Total MET-minutes	−0.04 (−0.047,−0.032), < 0.001	−0.044 (−0.052,−0.035), < 0.001
Pittsburgh sleep quality index	−0.005 (−0.l017, 0.007), 0.384	−0.028 (−0.040,−0.016), < 0.001
Sleep duration	−0.057 (−0.099,−0.017), 0.005	0.094 (0.056, 0.133), < 0.001
Self rating depression score	0.201 (0.154, 0.248), < 0.001	0.001 (0.001, 0.002), < 0.001
Self rating anxiety score	0.215 (0.165, 0.266), < 0.001	0.002 (0.001, 0.003), < 0.001
Fasting blood glucose	−0.142 (−0.181,−0.103), < 0.001	−0.228 (−0.273,−0.183), < 0.001
Alanine aminotransferase	0.045 (0.030, 0.060), < 0.001	−0.045 (−0.061,−0.029), < 0.001
Aspartate aminotransferase	0.026 (0.007, 0.046), 0.008	−0.028 (−0.049,−0.007), 0.008
Creatinine	−0.032 (−0.058,−0.006), 0.016	−0.049 (−0.71,−0.027), < 0.001
Total cholesterol	−0.095 (−0.124,−0.066), < 0.001	−0.146 (−0.176,−0.115), < 0.001
Triglyceride	0.013 (−0.001, 0.027), 0.066	−0.048 (−0.064,−0.032), < 0.001

**Table 5:** Univariate logistic regression analysis stratified by sex-specific study population (OR, 95%CI, P)

	Men	Women
< 45 years (vs. > 60 years)	1.28 (1.16, 1.41), < 0.001	2.08 (1.88, 2.29) ,<0.001
45–60 years (vs. > 60 years)	1.25 (1.12, 1.37), < 0.001	1.38 (1.24, 1.53), < 0.001
Neck circumference (≥ 40 vs. < 40 cm)	1.24 (1.16, 1.34), < 0.001	2.13 (1.61, 2.82), < 0.001
Abdominal obesity (yes vs. no)	1.26 (1.15, 1.32), < 0.001	0.79 (0.74, 0.85), < 0.001
Junior high (vs. ≤ primary)	1.24 (1.14, 1.35), < 0.001	1.33 (1.22, 1.44), < 0.001
≥Senior high (vs. ≤ primary)	1.99 (1.82, 2.19), < 0.001	2.12 (1.95, 2.30), < 0.001
Occupation (intelligent vs. manual)	1.22 (1.13, 1.31), < 0.001	1.52 (1.42, 1.63), < 0.001
Smoking ( yes vs. no )	0.80 (0.75, 0.86), < 0.001	1.02 (0.93, 1.12), 0.663
Alcohol intake (yes vs. no)	1.13 (1.05, 1.21), 0.001	1.50 (1.33, 1.69), < 0.001
Physical activity (middle vs. high)	1.32 (1.20, 1.44), < 0.001	1.49 (1.38, 1.61), < 0.001
Physical activity( low vs. high)	1.27 (1.14, 1.420, < 0.001	1.32 (1.19, 1.46), < 0.001
Sleep quality (poor vs. good)	0.98 (0.90, 1.06), 0.593	0.91 (0.85, 0.97), 0.007
Sleep duration (≤ 6 h vs. 6–7 h)	1.06 (0.95, 1.19), 0.299	0.88 (0.79, 0.96), 0.006
Sleep duration (> 8 h vs. 6–7 h)	0.95 (0.87, 1.03), 0.178	1.13 (1.01, 1.27), 0.037
Depression ( yes vs. no )	2.65 (1.97, 3.55), < 0.001	1.56 (1.32, 1.84), < 0.001
Anxiety (yes vs. no )	2.19 (1.76, 2.73), < 0.001	1.34 (1.18, 1.51), < 0.001
Hypertension ( yes vs. no )	0.84 (0.78, 0.91), < 0.001	0.74 (0.67, 0.81), < 0.001
Diabetes (yes vs. no )	0.84 (0.73, 0.96), 0.009	0.75 (0.65, 0.87), < 0.001
CVD (yes vs. no)	1.37 (0.99, 1.89), 0.061	0.87 (0.66, 1.15), 0.342
Dyslipidemia (yes vs. no)	1.08 (0.99, 1.17), 0.060	1.19 (1.09, 1.29), < 0.001
Renal inadequacy (yes vs. no)	1.03 (0.77, 1.38), 0.836	0.81 (0.69, 0.93), 0.004

Stratified analysis showed mainly consistent results with the above as given in Supplementary Tables 7–9.

### Principal component analysis

Obesity parameters (neck circumference, abdominal circumference), glucose metabolism (FPG), psychological status (anxiety status in men and women, depression status in men), BP, liver function (in men), lipid metabolism (TC and TG in men), sleep parameters (sleep quality in women), and renal function (in women) are the main factors associated with elevated PAC as in Tables 6, 7, 8 and 9.

**Table 6:** The component score coefficient matrix of men and women participants

	Component					
	1	2	3	4	5	6
Men	/	/	/	/	/	/
Self rating depression score	0.914	/	/	/	/	/
Self rating anxiety score	0.919	/	/	/	/	/
Alanine aminotransferase (µ/l)	/	0.923	/	/	/	/
Aspartate aminotransferase (µ/l)	/	0.932	/	/	/	/
Neck circumference (cm)	/	/	0.894	/	/	/
Abdominal circumference (cm)	/	/	0.860	/	/	/
Total cholesterol (mg/dl)	/	/	/	0.690	/	/
Triglyceride (mg/dl)	/	/	/	0.647	/	/
Fasting blood glucose (mmol/l)	/	/	/	0.693	/	/
Age (years)	/	/	/	/	0.827	/
Systolic blood pressure (mmHg)	/	/	/	/	0.691	/
Diastolic blood pressure (mmHg)	/	/	/	/	/	−0.842
Total MET-minutes	/	/	/	/	/	/
Women	/	/	/	/	/	/
Systolic blood pressure (mmHg)	0.909	/	/	/	/	/
Diastolic blood pressure (mmHg)	0.906	/	/	/	/	/
Neck circumference (cm)	/	0.885	/	/	/	/
Abdominal circumference (cm)	/	0.819	/	/	/	/
Self rating anxiety score	/	/	0.826	/	/	/
Pittsburgh sleep quality index	/	/	0.817	/	/	/
Fasting blood glucose (mmol/l)	/	/	/	0.707	/	/
Creatinine (umol /l )	/	/	/	0.611	/	/
Triglyceride (mg/dl)	/	/	/	/	/	/
Total MET-minutes	/	/	/	/	/	/

**Table 7:** Multivariate linear and logistic regression analysis between parameters and log plasma aldosterone concentration in sex-specific population with hypertension and or diabetes

	Men	Women
Multivariate linear regression	B (95% CI), P	B (95% CI), P
Age	/	−0.15 (−0.24,−0.06), 0.002
Neck circumference	0.78 (0.49, 1.06),<0.001	/
Abdominal circumference	−0.27 (−0.47,−0.07), 0.010	/
Educational level	0.05 (0.04, 0.06), < 0.001	0.03 (0.01, 0.04), < 0.001
Occupation	/	0.03 (0.01, 0.05), 0.007
Smoking	−0.06 (−0.08,−0.05),<0.001	/
Drinking	/	/
Systolic blood pressure	−0.45 (−0.61,−0.30), < 0.001	/
Diastolic blood pressure	0.19 (0.03, 0.35), 0.019	/
Total MET-minutes	/	−0.02 (−0.04,−0.01), 0.022
P1	2.98 (0.90, 5.06), 0.005	/
	/	/
Multivariate logistic regression	OR (95% CI), P	OR (95% CI), P
Abdominal obesity (yes vs. no)	1.27 (1.11, 1.46), 0.001	/
Junior high (vs. ≤ Primary)	1.53 (1.31, 1.79), < 0.001	1.11 (0.91, 1.36), 0.298
≥Senior high (vs. ≤ Primary)	2.23 (1.86, 2.67), < 0.001	1.59 (1.24, 2.05), < 0.001
Occupation (Intelligent vs. Manual)	/	1.25 (1.00, 1.56), 0.047
Smoking (yes vs. no)	0.63 (0.55, 0.72), < 0.001	/
Alcohol intake (yes vs. no)	/	/
Physical activity (middle vs. high)	/	/
Physical activity (low vs. high)	/	/
P1	/	/

**Table 8:** Multivariate linear and logistic regression analysis between parameters and log plasma aldosterone concentration in sex-specific population populations without hypertension and diabetes

	Men	Women
Multivariate linear regression	B (95% CI), P	B (95% CI), P
Age	/	/
Neck circumference	0.64 (0.44, 0.84), < 0.001	0.44 (0.25, 0.62), < 0.001
Abdominal circumference	−0.18 (−0.32,−0.04),0.012	−0.39 (−0.51,−0.27), < 0.001
Educational level	0.032 (0.025, 0.040), < 0.001	0.03 (0.02, 0.04), < 0.001
Occupation	/	0.02 (0.01, 0.04), < 0.001
Smoking	−0.04 (−0.05,−0.03), < 0.001	/
Drinking	/	0.03 (0.01, 0.05), 0.001
Systolic blood pressure	−0.51 (−0.67,−0.35), < 0.001	−0.20 (−0.32,−0.08), 0.001
Diastolic blood pressure	0.42 (0.28, 0.56), < 0.001	/
Total MET-minutes	−0.03 (−0.04,−0.02), < 0.001	−0.03 (−0.04,−0.01),<0.001
Pittsburgh sleep quality index	/	−0.03 (−0.04,−0.01), 0.002
Sleep duration (h)	−0.12 (−0.18,−0.06), < 0.001	/
Self rating depression score	0.22 (0.16, 0.29), < 0.001	/
Self rating anxiety score	/	0.15 (0.09, 0.22), < 0.001
Fasting blood glucose	−0.44 (−0.53,−0.34), < 0.001	−0.30 (−0.40,−0.21), < 0.001
Alanine aminotransferase	0.026 (0.002, 0.049), 0.033	−0.03 (−0.05,−0.01), 0.014
Aspartate aminotransferase	/	/
Creatinine	/	−0.04 (−0.08,−0.01),0.012
P1	/	/
Multivariate logistic regression	OR (95% CI), P	OR (95% CI), P
< 45 years (vs. > 60 years)	/	1.35 (1.11, 1.64), 0.003
45–60 years (vs. > 60 years)	/	1.09 (0.90, 1.33), 0.380
Neck circumference (≥ 40 vs. < 40 cm)	1.20 (1.07, 1.35), 0.002	1.85 (1.20, 2.84), 0.005
Abdominal obesity (yes vs. no)	1.14 (1.03, 1.27), 0.017	0.84 (0.76, 0.93), 0.001
Junior high (vs. ≤ primary)	1.14 (1.01, 1.29), 0.034	1.15 (1.02, 1.30), 0.027
≥Senior high (vs. ≤ primary)	1.74 (1.52, 1.99), < 0.001	1.70 (1.48, 1.95), < 0.001
Occupation (intelligent vs. manual)	/	1.13 (1.01, 1.26), 0.034
Smoking (yes vs. no)	0.79 (0.71, 0.88), < 0.001	
Alcohol intake (yes vs. no)	1.13 (1.01, 1.25), 0.026	1.39 (1.18, 1.63), < 0.001
Physical activity (middle vs. high)	1.16 (1.03, 1.31), 0.015	1.38 (1.25, 1.54), < 0.001
Physical activity (low vs. high)	1.09 (0.94, 1.27), 0.245	1.21 (1.05, 1.39), 0.008
Sleep duration (≤ 6 h vs. 6–7 h)	1.20 (1.03, 1.40), 0.017	/
Sleep duration (> 8 h vs. 6–7 h)	0.95 (0.86, 1.06), 0.341	/
Depression (yes vs. no)	/	1.50 (1.21, 1.86), < 0.001
Anxiety (yes vs. no)	1.87 (1.42, 2.47), < 0.001	/
P1	/	/

**Table 9:** Multivariate linear and logistic regression analysis between parameters and log plasma aldosterone concentration in sex-specific population without hypertension and diabetes and SDB.

	Men	Women
Multivariate linear regression	B (95% CI), P	B (95% CI), P
Age	/	/
Neck circumference	0.32 (0.09, 0.56), 0.007	0.46 (0.27, 0.66), < 0.001
Abdominal circumference	/	−0.38 (−0.51,−0.25), < 0.001
Educational level	0.03 (0.02, 0.04), < 0.001	0.03 (0.02, 0.04), < 0.001
Occupation	/	0.02 (0.01, 0.04), < 0.001
Smoking	−0.04 (−0.05,−0.03), < 0.001	/
Drinking	/	0.03 (0.02, 0.05), < 0.001
Systolic blood pressure	−0.63 (−0.81,−0.45),<0.001	−0.43 (−0.59,−0.28), < 0.001
Diastolic blood pressure	0.48 (0.32, 0.63), < 0.001	0.33 (0.18, 0.48), < 0.001
Total MET-minutes	−0.016 (−0.029,−0.004), 0.012	−0.03 (−0.04,−0.01), < 0.001
Pittsburgh sleep quality index	/	−0.02 (−0.04,−0.01), 0.009
Sleep duration (h)	−0.08 (−0.15,−0.01), 0.031	/
Self rating depression score	/	/
Self rating anxiety score	0.26 (0.17, 0.34), < 0.001	0.14 (0.07, 0.21), < 0.001
Fasting blood glucose	−0.42 (−0.53,−0.30), < 0.001	−0.29 (−0.39, 0.19), < 0.001
Alanine aminotransferase	/	−0.03 (−0.06,−0.01), 0.011
Aspartate aminotransferase	/	/
Creatinine	/	−0.04 (−0.08,−0.01), 0.015
P1	/	/
Multivariate logistic regression	OR (95% CI), P	OR (95% CI), P
< 45 years (vs. > 60 years)	/	1.31 (1.05, 1.63), 0.016
45–60 years (vs. > 60 years)	/	1.08 (0.87, 1.34),0.510
Neck circumference (≥ 40 vs. < 40 cm)	/	3.11 (1.48, 6.53), 0.003
Abdominal obesity (yes vs. no)	1.19 (1.03, 1.36), 0.016	0.89 (0.80, 0.99), 0.024
Junior high (vs. ≤ primary)	1.15 (0.99, 1.33), 0.067	1.15 (1.02, 1.30), 0.028
≥Senior high (vs. ≤ primary)	1.53 (1.30, 1.80), < 0.001	1.67 (1.45, 1.93), < 0.001
Occupation (intelligent vs. manual)	/	1.17 (1.05, 1.31), 0.006
Smoking (yes vs. no)	0.80 (0.71, 0.90), < 0.001	/
Alcohol intake (yes vs. no)	/	1.41 (1.19, 1.65), < 0.001
Physical activity (middle vs. high)	1.23 (1.05, 1.43), 0.008	1.35 (1.21, 1.50), < 0.001
Physical activity (low vs. high)	0.99 (0.82, 1.20), 0.916	1.20 (1.04, 1.38), 0.012
Sleep quality (poor vs. good)	/	/
Sleep duration (≤ 6 h vs. 6–7 h)	/	/
Sleep duration (> 8 h vs. 6–7 h)	/	/
Depression (yes vs. no)	/	1.56 (1.25, 1.94), < 0.001
Anxiety (yes vs. no)	1.83 (1.31, 2.56), < 0.001	/
P1	/	/

## Discussion

Current investigation is one of large-scale population-based studies to explore the factors associated with elevated PAC. Main results encompass: obesity parameters (neck circumference, abdominal circumference), glucose metabolism (FPG), psychological status (anxiety status in men and women, depression status in men), BP, liver function (in men), lipid metabolism (TC and TG in men), sleep parameters (sleep quality in women), renal function (in women) are the main factors associated with elevated PAC.

Elevated circulating aldosterone has consistently been shown to be a risk factor for vascular disease and renal dysfunction in various population [22] and might be a mediator between life style related factors and cardiovascular diseases [11]. Factors which cause elevation in circulating aldosterone are not clear, and studies suggest lifestyle-related factors be involved, whereas less studied. In addition, several studies have observed gender differences in levels and or regulators of aldosterone between men and women [5]. Previous several studies indicate that females have higher aldosterone than males, these sex differences are most likely caused by gonadal hormones [28]. Therefore, we used a cross-sectional design to explore the lifestyle-related factors associated with PAC in sex-specific larger sample community dwellers.

### Physical activity

Relationship between physical activity and changes in circulating aldosterone is inconclusive. In the present study, a negative correlation between PAC and physical activity was observed in both men and women participants. A few studies have reported a change in PAC with aerobic exercise training [29, 30]. Hespel et al. were among the first to report that in normotensive population, reductions in PAC were associated with an increase in physical work capacity with aerobic exercise training [29]. Braith et al. demonstrated that among patients with heart failure, PAC significantly decreased by approximately 50 pg ml − 1 after 16 weeks of aerobic exercise training [30]. Aerobic exercise training has been shown to suppress plasma renin activity, leading to a reduction in angiotensin II, which may be followed by decrease in aldosterone biosynthesis [15]. However, further mechanistic studies are needed.

### Accumulation of fat in body

In this study, a positive correlation was observed between abdominal circumference and PAC in men. Several studies show that aldosterone excess is often present in obesity and associated with obesity [31], possibly because adipose cells in adipose tissue possess enzyme aldosterone synthase, which independently produces aldosterone and stimulates the production of adrenal aldosterone-promoting secretions of hepatic origin [32]. Notably, we also observed a positive correlation between neck circumference and PAC in men and women. Neck circumference, a marker for determining upper body subcutaneous adipose tissue distribution, has also been considered an anthropometric indicator of obesity [33]. Previous studies have reported strong correlation between neck circumference and visceral adipose tissue [34]. Therefore, it is likely that people with larger neck circumference have high PAC mediated by subcutaneous adipose tissue.

### Alcohol intake

Consistent with previous studies, a positive correlation between alcohol consumption and aldosterone was observed in both men and women. The mechanism may be that the ethanol contained in alcohol reduces the expression of the nuclear receptor subfamily 3 group C member 2 gene associated with aldosterone synthesis and reduces the MR-mediated negative feedback [35]. This means that either abstinence or controlling alcohol consumption has an effect on controlling aldosterone levels.

### Sleep

In the present study, sleep duration is inversely associated with PAC in men. Evidence show that salivary aldosterone arousal response is influenced by sleep duration. Rhythmic variation in aldosterone is also associated with sleep duration, and wakefulness time [36]. However, no correlation between overall subjective sleep quality and PAC was observed, and the reason may lie in the subjectivity of the sleep quality scale.

### Mental state

Higher levels of PAC have been reported in depression [37], and anxiety in animal models [38]. This study also show that depression and anxiety status are positively associated with PAC. We also observed a positive correlation between education status, and mental work with PAC. It is likely that this is related to the RAAS activation induced by mental stress. This may indicate that maintaining a good mental state may have potential significance with maintaining normal PAC.

One of the differences of current study with previous ones is that we collected plasma samples for aldosterone measurements without requirement as in clinical setting. All blood samples were collected between fasting conditions (≥ 8 h) in the morning of local working hours and the procedure was < 30 min for most participants. However, consistent with previous studies, PAC appears to be higher in women than in men, although we did not perform statistical comparison on this part. Therefore, circulating PAC like this may reflect one’s real physiological levels for a certain duration of time, whereas not determined.

This study is strengthened by relatively large community dwellers, which allowed us to conduct sex-specific analysis. In addition, we included more life style related factors to acquire objective results. However, some limitations should also be taken into account. Importantly we failed to consider sodium and potassium, important regulators of aldosterone, and volume status, which may have brought bias to the results. However, the area where the study population live in is characterized by high sodium and low potassium intake intake [39] and therefore can be generalized to similar populations.

In conclusion, lower physical activity, alcohol consumption, higher BP, fat accumulation, dyslipidemia, higher FPG, and presence of depression and anxiety were the main factors associated with PAC.

## Supplementary Information

Supplementary Material 1.

## Data Availability

No datasets were generated or analysed during the current study.
