# Correction: Associated lifestyle factors of elevated plasma aldosterone concentration in community population, gender-stratified analysis of a cross-sectional survey

**DOI:** 10.1186/s12889-024-19323-x

**Published:** 2024-10-04

**Authors:** Adalaiti Maitituersun, Mulalibieke Heizhati, Nanfang Li, Lin Gan, Mei Li, Ling Yao, Wenbo Yang, Shasha Liu, Xiayire Aierken, Hui Wang, Miaomiao Liu, Jing Hong, Ting Wu, Delian Zhang, Qing Zhu

**Affiliations:** Hypertension Center of People’s Hospital of Xinjiang Uygur Autonomous RegionXinjiang Hypertension Institute, NHC Key Laboratory of Hypertension Clinical ResearchKey Laboratory of Xinjiang Uygur Autonomous Region “Hypertension Research Laboratory”, Xinjiang Clinical Medical Research Center for Hypertension (Cardio-Cerebrovascular) Diseases, No. 91 Tianchi Road, Urumqi, 830001 Xinjiang China

**Correction: BMC Public Health (2024) 24:1370**

10.1186/s12889-024-18796-0

The original publication of this article contained an incorrect affiliation. The incorrect and correct information is listed in this correction article.

Incorrect

NHC Key Laboratory of Hypertension Clinical Research, Key Laboratory of Xinjiang Uygur Autonomous Region, Hypertension Research Laboratory, Xinjiang Clinical Medical Research Center for Hypertension (Cardio-Cerebrovascular, Hypertension Center of People’s Hospital of Xinjiang Uygur Autonomous Region, Xinjiang Hypertension Institute, No. 91 Tianchi Road, Urumqi, 830001, Xinjiang, China

Correct

Hypertension Center of People’s Hospital of Xinjiang Uygur Autonomous RegionXinjiang Hypertension Institute; NHC Key Laboratory of Hypertension Clinical ResearchKey Laboratory of Xinjiang Uygur Autonomous Region “Hypertension Research Laboratory”; Xinjiang Clinical Medical Research Center for Hypertension (Cardio-Cerebrovascular) Diseases

