# Supplementary material for: Associated lifestyle factors of elevated plasma aldosterone concentration in community population, gender-stratified analysis of a cross-sectional survey
Source: BMC Public Health. 2024 May 22;24:1370. doi: 10.1186/s12889-024-18796-0 (PMC11110359; doi:10.1186/s12889-024-18796-0)
Supplement: Supplementary file 1 — Supplementary Material 1. [file 12889_2024_18796_MOESM1_ESM.docx]

| Table 7. Multivariate linear regression analysis between parameters and log plasma aldosterone concentration in gender-specific population with hypertension and or diabetes. | | | | | | |
| --- | --- | --- | --- | --- | --- | --- |
|  | Men | | | Women | | |
|  | B | 95% CI | P | B | 95% CI | P |
| Log age |  |  |  | -0.11 | -0.22, -0.01 | 0.035 |
| Log neck circumference | 0.77 | 0.49, 1.06 | <0.001 |  |  |  |
| Log abdominal circumference | -0.27 | -0.47, -0.06 | 0.011 |  |  |  |
| Educational level | 0.05 | 0.04, 0.06 | <0.001 | 0.03 | 0.01, 0.04 | <0.001 |
| Occupation |  |  |  | 0.04 | 0.01, 0.07 | 0.002 |
| Smoking | -0.06 | -0.07, -0.04 | <0.001 |  |  |  |
| Drinking |  |  |  |  |  |  |
| Log systolic blood pressure | -0.46 | -0.62, -0.31 | <0.001 |  |  |  |
| Log diastolic blood pressure | 0.21 | 0.05, 0.37 | 0.011 |  |  |  |
| Log total MET-minutes | -0.02 | -0.03, 0.00 | 0.048 | -0.03 | -0.05, -0.01 | 0.006 |
| Log Pittsburgh sleep quality index |  |  |  |  |  |  |
| Log sleep duration (h) |  |  |  |  |  |  |
| Log self rating depression score |  |  |  |  |  |  |
| Log self rating anxiety score |  |  |  |  |  |  |
| Log fasting blood glucose |  |  |  |  |  |  |
| Log alanine aminotransferase |  |  |  |  |  |  |
| Log aspartate aminotransferase |  |  |  |  |  |  |
| Log creatinine |  |  |  |  |  |  |
| Log total cholesterol |  |  |  |  |  |  |
| Log triglyceride |  |  |  |  |  |  |
|  | | | |  | | |

| Table8. Multivariate logistic regression analysis stratified by gender-specific study population with hypertension and or diabetes. | | | | | | |
| --- | --- | --- | --- | --- | --- | --- |
|  | Men | | | Women | | |
|  | OR | 95% CI | P | OR | 95% CI | P |
| <45 years (vs >60 years) |  |  |  |  |  |  |
| 45-60 years (vs >60 years) |  |  |  |  |  |  |
| Neck circumference ( ≥40 vs <40cm) |  |  |  |  |  |  |
| Abdominal obesity (men≥90 vs ＜90，women≥85 vs ＜85) | 1.29 | 1.12, 1.50 | <0.001 |  |  |  |
| Junior high (vs ≤Primary) | 1.48 | 1.26, 1.74 | <0.001 | 1.12 | 0.91, 1.39 | 0.296 |
| ≥Senior high (vs ≤Primary) | 2.22 | 1.84, 2.69 | <0.001 | 1.52 | 1.14, 2.01 | 0.004 |
| Occupation (Intelligent vs Manual) |  |  |  | 1.40 | 1.09, 1.80 | 0.009 |
| Smoking ( yes vs no ) | 0.64 | 0.56, 0.74 | <0.001 |  |  |  |
| Alcohol intake (yes vs no) |  |  |  |  |  |  |
| Physical activity (middle vs high) |  |  |  | 1.28 | 1.03, 1.59 | 0.026 |
| Physical activity( low vs high) |  |  |  | 1.37 | 1.03, 1.81 | 0.032 |
| sleep quality (poor vs good） |  |  |  |  |  |  |
| Sleep duration (≤6h vs 6-7 h) |  |  |  |  |  |  |
| Sleep duration (>8h vs 6-7 h) |  |  |  |  |  |  |
| Depression ( yes vs no ) |  |  |  |  |  |  |
| Anxiety (yes vs no ) |  |  |  |  |  |  |
| Hypertension ( yes vs no ) |  |  |  |  |  |  |
| Diabetes (yes vs no ) |  |  |  |  |  |  |
| CVD (yes vs no) |  |  |  |  |  |  |
| Renal inadequacy (yes vs no) |  |  |  |  |  |  |
|  | | | | | | |

| Table9. Multivariate linear regression analysis between parameters and log plasma aldosterone concentration in gender-specific population populations without hypertension and diabetes. | | | | | | |
| --- | --- | --- | --- | --- | --- | --- |
|  | Men | | | Women | | |
|  | B | 95% CI | P | B | 95% CI | P |
| Log age |  |  |  |  |  |  |
| Log neck circumference | 0.64 | 0.44, 0.84 | <0.001 | 0.43 | 0.24, 0.61 | <0.001 |
| Log abdominal circumference | -0.18 | -0.32, -0.04 | 0.012 | -0.37 | -0.50, -0.25 | <0.001 |
| Educational level | 0.033 | 0.025, 0.040 | <0.001 | 0.03 | 0.02, 0.04 | <0.001 |
| Occupation |  |  |  | 0.022 | 0.009, 0.035 | 0.001 |
| Smoking | -0.04 | -0.05, -0.03 | <0.001 |  |  |  |
| Drinking | 0.012 | 0.001, 0.024 | 0.039 | 0.04 | 0.02, 0.06 | <0.001 |
| Log systolic blood pressure | -0.50 | -0.66, -0.34 | <0.001 | -0.23 | -0.35, -0.10 | <0.001 |
| Log diastolic blood pressure | 0.42 | 0.28, 0.56 | <0.001 |  |  |  |
| Log total MET-minutes | -0.03 | -0.04, -0.02 | <0.001 | -0.03 | -0.04, -0.01 | <0.001 |
| Log Pittsburgh sleep quality index |  |  |  | -0.03 | -0.05, -0.01 | 0.002 |
| Log sleep duration (h) | -0.12 | -0.18, -0.06 | <0.001 |  |  |  |
| Log self rating depression score | 0.22 | 0.16, 0.29 | <0.001 |  |  |  |
| Log self rating anxiety score |  |  |  | 0.14 | 0.07, 0.20 | <0.001 |
| Log fasting blood glucose | -0.04 | -0.05, -0.03 | <0.001 | -0.27 | -0.37, -0.02 | <0.001 |
| Log alanine aminotransferase | 0.027 | 0.003, 0.051 | 0.026 | -0.03 | -0.05, -0.01 | 0.022 |
| Log aspartate aminotransferase |  |  |  |  |  |  |
| Log creatinine |  |  |  | -0.04 | -0.08, -0.01 | 0.014 |
| Log total cholesterol |  |  |  |  |  |  |
| Log triglyceride |  |  |  |  |  |  |
|  | | | |  | | |

| Table10. Multivariate logistic regression analysis stratified by gender-specific study population without hypertension and diabetes. | | | | | | |
| --- | --- | --- | --- | --- | --- | --- |
|  | Men | | | Women | | |
|  | OR | 95% CI | P | OR | 95% CI | P |
| <45 years (vs >60 years) |  |  |  | 1.31 | 1.08, 1.60 | 0.007 |
| 45-60 years (vs >60 years) |  |  |  | 1.08 | 0.89, 1.32 | 0.449 |
| Neck circumference ( ≥40 vs <40cm) | 1.19 | 1.07, 1.35 | 0.002 | 1.87 | 1.21, 2.89 | 0.005 |
| Abdominal obesity (men≥90 vs ＜90，women≥85 vs ＜85) | 1.15 | 1.03, 1.28 | 0.012 | 0.84 | 0.76, 0.93 | 0.001 |
| Junior high (vs ≤Primary) | 1.15 | 1.02, 1.29 | 0.028 | 1.14 | 1.00, 1.29 | 0.043 |
| ≥Senior high (vs ≤Primary) | 1.74 | 1.52, 1.99 | <0.001 | 1.69 | 1.48, 1.95 | <0.001 |
| Occupation (Intelligent vs Manual) |  |  |  | 1.12 | 1.01, 1.25 | 0.040 |
| Smoking ( yes vs no ) | 0.79 | 0.72, 0.89 | <0.001 |  |  |  |
| Alcohol intake (yes vs no) | 1.12 | 1.01, 1.25 | 0.031 | 1.39 | 1.19, 1.64 | <0.001 |
| Physical activity (middle vs high) | 1.17 | 1.04, 1.32 | 0.012 | 1.38 | 1.24, 1.54 | <0.001 |
| Physical activity( low vs high) | 1.10 | 0.95, 1.28 | 0.201 | 1.19 | 1.04, 1.38 | 0.011 |
| sleep quality (poor vs good） |  |  |  |  |  |  |
| Sleep duration (≤6h vs 6-7 h) | 1.20 | 1.03, 1.40 | 0.019 |  |  |  |
| Sleep duration (>8h vs 6-7 h) | 0.95 | 0.85, 1.05 | 0.312 |  |  |  |
| Depression ( yes vs no ) |  |  |  | 1.48 | 1.19, 1.84 | <0.001 |
| Anxiety (yes vs no ) | 1.83 | 1.39, 2.43 | <0.001 |  |  |  |
| Hypertension ( yes vs no ) |  |  |  |  |  |  |
| Diabetes (yes vs no ) |  |  |  |  |  |  |
| CVD (yes vs no) |  |  |  |  |  |  |
| Renal inadequacy (yes vs no) |  |  |  |  |  |  |
|  | | | | | | |

| Table11. Multivariate linear regression analysis between parameters and log plasma aldosterone concentration in gender-specific population without hypertension and diabetes and with SDB. | | | | | | |
| --- | --- | --- | --- | --- | --- | --- |
|  | Men | | | Women | | |
|  | B | 95% CI | P | B | 95% CI | P |
| Log age | 0.09 | 0.02, 0.18 | 0.019 | -0.29 | -0.54, -0.03 | 0.029 |
| Log neck circumference | 0.05 | 0.20, 0.77 | 0.001 |  |  |  |
| Log abdominal circumference |  |  |  |  |  |  |
| Educational level | 0.05 | 0.03, 0.06 | <0.001 | 0.04 | 0.01, 0.06 | 0.005 |
| Occupation | 0.027 | 0.002, 0.051 | 0.032 |  |  |  |
| Smoking |  |  |  |  |  |  |
| Drinking |  |  |  |  |  |  |
| Log systolic blood pressure |  |  |  |  |  |  |
| Log diastolic blood pressure |  |  |  |  |  |  |
| Log total MET-minutes | -0.04 | -0.06, -0.02 | <0.001 |  |  |  |
| Log Pittsburgh sleep quality index |  |  |  |  |  |  |
| Log sleep duration (h) | -0.15 | -0.26, -0.04 | 0.010 |  |  |  |
| Log self rating depression score | 0.17 | 0.05, 0.30 | 0.006 |  |  |  |
| Log self rating anxiety score |  |  |  |  |  |  |
| Log fasting blood glucose | -0.47 | -0.64, -0.29 | <0.001 |  |  |  |
| Log alanine aminotransferase |  |  |  |  |  |  |
| Log aspartate aminotransferase |  |  |  |  |  |  |
| Log creatinine | -0.076 | -0.147, -0.004 | 0.038 |  |  |  |
| Log total cholesterol |  |  |  |  |  |  |
| Log triglyceride |  |  |  |  |  |  |
|  | | | |  | | |

| Table12. Multivariate logistic regression analysis stratified by gender-specific study population without hypertension and diabetes and with SDB. | | | | | | |
| --- | --- | --- | --- | --- | --- | --- |
|  | Men | | | Women | | |
|  | OR | 95% CI | P | OR | 95% CI | P |
| <45 years (vs >60 years) |  |  |  |  |  |  |
| 45-60 years (vs >60 years) |  |  |  |  |  |  |
| Neck circumference ( ≥40 vs <40cm) | 1.32 | 1.04, 1.66 | 0.022 |  |  |  |
| Abdominal obesity (men≥90 vs ＜90，women≥85 vs ＜85) |  |  |  |  |  |  |
| Junior high (vs ≤Primary) | 1.18 | 0.92, 1.51 | 0.195 |  |  |  |
| ≥Senior high (vs ≤Primary) | 2.53 | 1.92, 3.33 | <0.001 |  |  |  |
| Occupation (Intelligent vs Manual) |  |  |  |  |  |  |
| Smoking ( yes vs no ) |  |  |  |  |  |  |
| Alcohol intake (yes vs no) | 1.38 | 1.12, 1.71 | 0.003 |  |  |  |
| Physical activity (middle vs high) | 1.29 | 1.03, 1.65 | 0.030 |  |  |  |
| Physical activity( low vs high) | 1.32 | 0.99, 1.74 | 0.058 |  |  |  |
| sleep quality (poor vs good） |  |  |  |  |  |  |
| Sleep duration (≤6h vs 6-7 h) |  |  |  |  |  |  |
| Sleep duration (>8h vs 6-7 h) |  |  |  |  |  |  |
| Depression ( yes vs no ) | 2.08 | 1.03, 4.19 | 0.041 |  |  |  |
| Anxiety (yes vs no ) |  |  |  |  |  |  |
| Hypertension ( yes vs no ) |  |  |  |  |  |  |
| Diabetes (yes vs no ) |  |  |  |  |  |  |
| CVD (yes vs no) |  |  |  |  |  |  |
| Renal inadequacy (yes vs no) |  |  |  |  |  |  |
|  | | | | | | |

| Table13. Multivariate linear regression analysis between parameters and log plasma aldosterone concentration in gender-specific population without hypertension and diabetes and SDB. | | | | | | |
| --- | --- | --- | --- | --- | --- | --- |
|  | Men | | | Women | | |
|  | B | 95% CI | P | B | 95% CI | P |
| Log age |  |  |  |  |  |  |
| Log neck circumference | 0.33 | 0.09, 0.56 | 0.006 | 0.46 | 0.27, 0.66 | <0.001 |
| Log abdominal circumference |  |  |  | -0.38 | -0.51, -0.25 | <0.001 |
| Educational level | 0.03 | 0.02, 0.04 | <0.001 | 0.03 | 0.02, 0.04 | <0.001 |
| Occupation |  |  |  | 0.02 | 0.01, 0.04 | <0.001 |
| Smoking | -0.04 | -0.05, -0.03 | <0.001 |  |  |  |
| Drinking |  |  |  | 0.03 | 0.02, 0.05 | <0.001 |
| Log systolic blood pressure | -0.63 | -0.81, -0.44 | <0.001 | -0.43 | -0.59, -0.28 | <0.001 |
| Log diastolic blood pressure | 0.47 | 0.31, 0.63 | <0.001 | 0.33 | 0.18, 0.48 | <0.001 |
| Log total MET-minutes | -0.017 | -0.029, -0.004 | 0.011 | -0.03 | -0.04, -0.01 | <0.001 |
| Log Pittsburgh sleep quality index |  |  |  | -0.02 | -0.04, -0.01 | 0.009 |
| Log sleep duration (h) | -0.08 | -0.15, -0.01 | 0.034 |  |  |  |
| Log self rating depression score |  |  |  |  |  |  |
| Log self rating anxiety score | 0.25 | 0.17, 0.34 | <0.001 | 0.14 | 0.07, 0.21 | <0.001 |
| Log fasting blood glucose | -0.42 | -0.53, -0.30 | <0.001 | -0.29 | -0.39, 0.19 | <0.001 |
| Log alanine aminotransferase |  |  |  | -0.03 | -0.06, -0.01 | 0.011 |
| Log aspartate aminotransferase |  |  |  |  |  |  |
| Log creatinine |  |  |  | -0.04 | -0.08, -0.01 | 0.015 |
| Log total cholesterol |  |  |  |  |  |  |
| Log triglyceride |  |  |  |  |  |  |
|  | | | |  | | |

| Table14. Multivariate logistic regression analysis stratified by gender-specific study population without hypertension and diabetes and SDB. | | | | | | |
| --- | --- | --- | --- | --- | --- | --- |
|  | Men | | | Women | | |
|  | OR | 95% CI | P | OR | 95% CI | P |
| <45 years (vs >60 years) |  |  |  | 1.31 | 1.05, 1.63 | 0.016 |
| 45-60 years (vs >60 years) |  |  |  | 1.08 | 0.87, 1.34 | 0.510 |
| Neck circumference ( ≥40 vs <40cm) |  |  |  | 3.11 | 1.48, 6.53 | 0.003 |
| Abdominal obesity (men≥90 vs ＜90，women≥85 vs ＜85) | 1.19 | 1.04, 1.36 | 0.015 | 0.89 | 0.80, 0.99 | 0.024 |
| Junior high (vs ≤Primary) | 1.15 | 0.99, 1.33 | 0.066 | 1.15 | 1.02, 1.30 | 0.028 |
| ≥Senior high (vs ≤Primary) | 1.53 | 1.30, 1.79 | <0.001 | 1.67 | 1.45, 1.93 | <0.001 |
| Occupation (Intelligent vs Manual) |  |  |  | 1.17 | 1.05, 1.31 | 0.006 |
| Smoking ( yes vs no ) | 0.80 | 0.71, 0.90 | <0.001 |  |  |  |
| Alcohol intake (yes vs no) |  |  |  | 1.41 | 1.19, 1.65 | <0.001 |
| Physical activity (middle vs high) | 1.23 | 1.06, 1.43 | 0.007 | 1.35 | 1.21, 1.50 | <0.001 |
| Physical activity( low vs high) | 0.99 | 0.82, 1.20 | 0.963 | 1.20 | 1.04, 1.38 | 0.012 |
| sleep quality (poor vs good） |  |  |  |  |  |  |
| Sleep duration (≤6h vs 6-7 h) |  |  |  |  |  |  |
| Sleep duration (>8h vs 6-7 h) |  |  |  |  |  |  |
| Depression ( yes vs no ) |  |  |  | 1.56 | 1.25, 1.94 | <0.001 |
| Anxiety (yes vs no ) | 1.81 | 1.29, 2.54 | 0.001 |  |  |  |
| Hypertension ( yes vs no ) |  |  |  |  |  |  |
| Diabetes (yes vs no ) |  |  |  |  |  |  |
| CVD (yes vs no) |  |  |  |  |  |  |
| Renal inadequacy (yes vs no) |  |  |  |  |  |  |
|  | | | | | | |
